# Food hypersensitivity-induced chronic gastrointestinal inflammation in a non-human primate model of diet-induced obesity

**DOI:** 10.1371/journal.pone.0214621

**Published:** 2019-04-04

**Authors:** Tomris Mustafa, Qun Li, Lauren E. Kelly, Anne Gibbon, Irwin Ryan, Keisha Roffey, Stephanie Simonds, Michael A. Cowley, Mark W. Sleeman

**Affiliations:** 1 Metabolism, Diabetes and Obesity Program, Biomedicine Discovery Institute, Monash University, Victoria, Australia; 2 Department of Physiology, Monash University, Victoria, Australia; 3 Monash Animal Research Platform, Monash University, Clayton, Victoria, Australia; Auburn University College of Veterinary Medicine, UNITED STATES

## Abstract

Experimental non-human primate models of obesity are induced through the introduction of atypically calorically rich diets. Studies in captive-bred macaques show the development of obesity and diabetes with similar complications to humans including eye and kidney diseases, nerve damage associated with pain and blood vessel damage. Diets differ in outcomes and here we document inflammation of the gastrointestinal tract that can be exacerbated through these dietary interventions. Following baseline physiological evaluation of body composition, Southern pigtail macaques were given a high-fat diet (HFD) for three months. This HFD consisted of lard, grains (including gluten), dairy and fructose that was otherwise omitted from a standard macaque diet (Chow). Physiological parameters were then reassessed before animals were reverted back to standard Chow for a further three months (remission). Consumption of the HFD resulted in food-mediated hypersensitivity marked by chronic weight loss, alopecia, malabsorption, protein-losing enteropathy and gross diffuse intestinal villi atrophy and lamina propria hypertrophy. Physiological changes were more highly pronounced in female macaques suggesting sex-specific differences but could be fully reversed through change of diet. Care should be taken in choosing non-human primate HFD diets for creating experimental models of obesity because they can induce severe food-driven chronic inflammation of the gastrointestinal tract that can eventuate to diet-induced chronic wasting and mortality.

## Introduction

Research with non-human primates (NHPs) has led to critical health related advances in treating human disease. Obesity and diabetes develops naturally in NHPs and this has been used to study the developmental nature and pathophysiological changes based on their genetic similarities to humans [1, 2]. There is a paucity of NHP research in this area as it is highly regulated and costly, and as a result much of the research has been to support pharmaceutical drug discovery, pre-clinical testing and optimisation of new novel therapeutics. As with humans, a captive-breed macaque has a long life-cycle, and so the onset of symptomologies can take at any time, up to 15 years to develop [2] and there is also always going to be limited animals with the same phenotypic profile at any one time. An additional contributing issue is lack of predictive markers for the ensuing metabolic disturbances. Although this natural model of obesity is highly relevant to both human physiology and pathology, experimental models of obesity with high caloric diets are now routinely being used to accelerate the development of disease phenotype (adiposity, insulin-resistance, dyslipidemia and metabolic syndrome)[3, 4].

Since a positive correlation exists between dietary fat consumption, weight gain and obesity in humans and many experimental animals [5], we have developed a model of obesity in macaques based around the use of a “hyper caloric westernised” diet. Previous work using primate diets formulated with saturated animal fats, cholesterol, and high-glycaemic carbohydrates referred to as the Typical American Diet (TAD) has shown a net final total energy intake of 30–50% fat; this leads to significantly accelerated adiposity, insulin resistance and dyslipidemia when fed to a macaque [4]. Work by Bremer and colleagues demonstrated high-fructose consumption, provided in the form of daily 15% fructose sweetened beverages, also resulted in rapid development of adiposity, insulin resistance, metabolic syndrome and overt type 2 diabetes [6, 7]. This approach now combined with the aforementioned westernised high-fat diet, is the most common method used to mitigate rapid-onset obesity in non-human primates [8, 9].

Captive-bred and maintained macaque colonies are however prone to the development of gastrointestinal disorders [10]. In particular **i**diopathic **c**hronic **e**nterocolitis (ICE) can account for up to 17% of all reported deaths [10–13]. Chronic episodes of loose stool, weight loss, poor body condition, malabsorption, exudative enteropathy with characteristic histopathological colonic crypt, epithelial and lamina propria characterises the condition [10, 11]. Although novel helicobacter species have been isolated and implicated in the pathogenesis of chronic idiopathic colitis [14], the causes of ICE in macaque colonies remains largely unknown and in most cases does not involve helicobacter or other known pathogens (such as Giardia, Salmonella, Shingella, Campylobacter and Yersinia). Previous studies have also suggested macaques are prone to food sensitivities that have an underlying immune component with parallels to ICE pathology. Rhesus macaques fed a high-gluten diet were shown to develop symptomologies consistent with celiac disease and chronic colitis [15, 16]. Similar findings were also reported in captive callitrichid in which animals developed severe immune reactions to dietary wheat proteins [17].

We have shown that variations in the high-fat diet (HFD) routinely used in these types of studies can lead to an immune-mediated hypersensitivity response in macaques that results in life threatening chronic weight loss, malabsorption, protein-losing enteropathy, alopecia and gastrointestinal inflammation, in a sex-specific manner. The clinical features of diet-induced gastroenteropathy including body wasting and hair loss could be fully recovered by eliminating the HFD diet. Careful attention should be paid to the formulation these diets and introducing novel macronutrients sources to captive-bred macaque colonies in studies involving dietary manipulations and interventions.

## Materials and methods

### Ethical statement

All protocols described involving the care and use of NHPs were approved by the Monash University Animal Ethics Committee (AEC) in accordance with the *Australian Code for the Care and Use of Animals for Scientific Purposes*, *8*^*th*^ *Edition*, *2013* and the *National Health and Medical Research Council Principles and Guidelines for the Care and Use of Non-Human Primates for Scientific Purposes*, *2016*. Health and wellbeing monitoring of the animals was carried out in cage on a daily basis or on handling under anaesthesia by trained animal care staff according to the NHP monitoring guidelines defined by the AEC which is based on a clinical observation scoring system that takes into consideration changes in coat condition, activity, movement/gait, behaviour, eating, drinking and alertness or body condition, body weight, body temperature, dehydration, mucous membrane colour, breathing in addition to the condition of eyes, teeth, tail, nose and extremities on handling. An experimental endpoint was reached if animals presented with any acute or chronic condition that impacted the welfare of the animal such as >15% chronic weight loss as defined under the NHP monitoring guidelines. Euthanasia was performed by first anaesthetising animals prior to administering a lethal dose of pentobarbital according to the procedures described below. To alleviate stress, discomfort and any pain caused by the described studies, animals were fully anaesthetised to assess body composition, weight or conduct blood withdrawals. All animals remaining at the end of the studies were either returned to the colony for breeding or have been repurposed for similar studies using an alternative custom high-fat diet to induce a NHP model of obesity.

### Southern pig-tail macaques

The 20 male and 20 female captive-bred Southern pigtail macaques (*Macacca nemestrina*), aged between 4–18 years selected for the studies were procured from the National Non-Human Primate Research and Breeding Facility (Monash University). Prior to selection, all animals underwent health screening, and had normal haematology and biochemical blood profiles with typical age-dependent body weight according to facility standards. Animals were housed in single-sex social groups of 5–10 animals in customised double Flex-A-Gon cages (Britz and Company, USA). Each housing unit consisted of ~25 square meter floor space and 2.5 meters of vertical height equipped with multiple automatic water nozzles, resting benches, fruit chute feeders, roller panels, dry food feeders, water/dry foragers, hanging barrel tunnels and multiple hanging wooden planks. Animals were also provided with daily access to outdoor enclosures of equivalent size. Environmental enrichment was provided in the form of fruit ice cups, cartons containing jelly, PVC tubes filled with fruits and nuts, paint rollers covered in treacle and seeds, foraging balls, muffins, wood shavings with hidden treats, chew sticks, puzzle treat dispensers, herb and perfume sprays, mirrors and plant foliage and alternated on a daily basis.

### Feeding regimen and diet composition

All Southern pigtail macaques at the facility were fed a nutritionally balanced diet that consisted of standard primate pellets (Chow) (Gordons Specialty Feeds, Australia) once daily (200g/macaque) with an energy density of 13Mj/kg (21% protein, 5.3% fibre, and 8% fat). All animals also received daily seasonal fruits and vegetables (300g/animal) in addition to food items provided as part of the enrichment program (e.g., dried fruit, seeds, nuts, maize, jelly, fruit juice, treacle, muffins, popcorn, meal worms, coconut flakes, plant foliage). The High Fat Diet (HFD) (Speciality Feeds, Australia) used in these studies was formulated and locally manufactured according to the Purina TAD diet with only minor variations from the original source with an energy density of 14.6 Mj/kg (12% protein, 13.7% fibre, 13% fat and 5.54% fructose). The HFD was provided as described above in addition to all the regular food enrichments. A recovery diet (Chow-R) consisted of double the daily allowance of Chow, fruits and vegetables in addition to protein supplementation in the form of eggs (2 eggs/animal/day). The source, formulation and calculated nutritional parameters of all diets used in the studies are listed in Table 1. All animals were given ad libitum access to water.

**Table 1:** The source, formulation and calculated nutritional parameters of the standard primate diet (Chow) and custom high-fat diet (HFD) compared to the Typical American diet (TAD) primate diet.

**Standard Primate Diet (Chow)**		**High-Fat Diet (HFD)**	
Gordons Specialty Feeds (Australia)		Specialty Feeds (Australia)	
Custom Diet		Custom Diet	
**Calculated Nutritional Parameters**	**%**	**Calculated Nutritional Parameters**	**%**
Protein	21	Protein	12
Total Fat	8	Total Fat	13
Fructose	-	Fructose	5.54
Crude Fibre	5.3	Crude Fibre	13.7
Acid Detergent Fibre	4.6	Acid Detergent Fibre	4.3
Digestible Energy	13 Mj/kg	Digestible Energy	14.6 Mj/kg
**Ingredients**		**Ingredients**	
Corn, sorghum, rice, lupins, soy protein, coconut meal, dried milk products, molasses, vegetable oil, sugar beet pulp, yeast, calcium carbonate, salt, vitamin and mineral premix, vitamin C (stabilised), vitamin E and vitamin D3.		Wheat, mill mix (bran and pollard), skim milk, sucrose, beef tallow, canola oil, guar gum, fructose, maize, cellulose, fish oil, casein, lard, calcium carbonate, salt, sulphur, vitamin C, potassium chloride, vitamin/mineral mix.	

### Dietary interventions

*Months (0–3)*; Following the completion of the procedures described below, a total of 10 male and 10 female macaques were randomly assigned to the HFD group. Animals were introduced to the HFD over a two-week period by first mixing the HFD with Chow. Once it was established that the macaques found the diet palatable with no immediate adverse effects, each animal was maintained fully on the HFD for 3 months. The remaining 10 male and 10 females assigned to the control diet group remained on regular Chow. *Months (3–6);* Following completion of the procedures, animals in the HFD group were switched to the recovery diet and remained on this diet for a further 3 months. All Chow and HFD animals were revaluated following this 3 month recovery period according to the standard procedures.

### Blood collections and procedures to evaluate body weight and metabolic parameters

All animals fasted overnight received a single intramuscular injection of an anaesthetic combination consisting of an equal mixture of tiletamine/zolazepam (1.4–3.2 mg/kg Zoletil100; Virbac Animal Health Australia) combined with butorphanol (0.1–0.3mg/kg; Butorphanol Tartrate (Butrogesic), Troy Laboratories Australia) and atropine (0.02–0.04mg/kg; Atrophine Sulphate (Atrosite (Illium)), Troy Laboratories Australia) and subsequently administered a partial reversal agent, flumazenil (0.01mg/kg; Hospira, Pfizer Australia) intramuscularly at the end of the procedures. Once immobilised and transferred to the procedure room, animals were initially maintained on 2–4% sevoflurane in 100% oxygen via face mask until a sufficient depth of anaesthesia (medium) was achieved. The animals were then intubated with a cuffed endotracheal tube and maintained on 1–2% sevoflurane for the entire duration of the procedures. For body composition determinations, anesthetised animals were transferred to a Dual X-Ray Absorptiometry (DEXA) (Horizon–A Hologic) and scanned in a prone position. A 23G X3/4” winged infusion needle was inserted into the gastrocnemius saphenous vein and blood was collected into EDTA Vacutainer tubes (Becton and Dickinson, USA) or SST Serum Separation Tubes (Becton and Dickinson, USA) and processed according to manufactures instructions. All procedures were conducted under aseptic conditions and depth of anaesthesia (muscle tone, palpebral reflexes, pedal reflexes) and physiological parameters (oxygen saturation rates, respiration and heart rate, body temperature) were continuously monitored throughout out the entire duration of experiments. Plasma and/or serum was used for analysis of haematology and serum serology by Gribbles Veterinary Pathology Services (Healthscope, Australia).

### Post-mortem tissue collections

Macaques that displayed greater than 15% total body weight loss or reached any other experimental endpoint as predefined by the Monash University Animal Ethics Committee (AEC) were euthanised. These animals were first anaesthetised, whole blood was collected and processed for biochemical profiles and serology prior to the intravenous administration of a lethal dose (0.5ml/kg body weight) of pentobarbital (Lethabarb; Virbac Pty Ltd., Australia). Representative tissue from sections of the gastrointestinal (GI) tract in addition to the heart, liver, kidneys and skin were then dissected and fixed in 10% formalin for subsequent gross histopathological examination. Stool samples were also collected and analysed for parasites and occult blood. Haematology, serum serology, stool analysis and preliminary histopathology was conducted by Gribbles Veterinary Pathology Services (Healthscope, Australia).

### Histopathology

Post-mortem samples of stomach, small intestine and colon collected from the three HFD-female macaques euthanised were fixed in 10% neutral buffered formalin, paraffin embedded and sectioned at 5μm. Additional GI tissues from Chow-fed control macaques that were euthanised in unrelated experiments were independently collected and processed under near identical conditions (three southern pigtail macaques). All sections were then stained at the same time with Hematoxylin and Eosin (H&E) or Giemsa May-Grünwald (MG) and viewed under a light microscope using a 5-40x objective. Giemsa May-Grünwald staining was carried out to allow the identification and quantitation of GI tissue eosinophils. Representative sections obtained from Chow- and HFD- groups were examined for gross histopathological changes; villus blunting and atrophy, crypt hyperplasia, inflammatory cell infiltration of the GI wall and changes in intestinal lymphatic tissues. A protocol to quantitate the total number of eosinophils in MG stained intestinal sections of Chow- and HFD- tissues was devised counting the total number of eosinophils positive cells over two adjacent villi including the basal zone.

### Immunohistochemical staining and imaging

Formalin-fixed and paraffin-embedded tissue sections (5μm thickness) mounted on glass microscope slides were immunohistochemically stained. Briefly, sections were deparaffinised and incubated in 0.1M citrate buffer (pH 6.1) at 98°C for 30 minutes using the DAKO PT Link system (Agilent Technologies, USA) for antigen retrieval. The sections were then stained with one or more of the following primary antibodies against immune cell markers at the specified dilution factors; *CD3* -marker for T cells (Rabbit Anti-CD, Dako-A0542, 1:100); *CD8-* marker for cytotoxic T-cells (Rabbit anti-CD8, Abcam- AB4055, 1:500); *CD68-* marker for macrophages **(**Rabbit anti-CD68, Atlas Antibodies- HPA048982, 1:1000); *CD11C-* marker for dendritic cells, monocytes/macrophages, neutrophils and natural killer cells **(**Rabbit anti-CD11C, Proteintech-60258-1, 1:200) and *NCAM1/CD56*-marker for natural killer cells, T cell subtypes and monocytes (Rabbit anti-NCAM1/CD56, Proteintech- 14255-1-AP, 1:500). This was followed by horse radish peroxidase labelled polymer secondary antibodies using the DAKO EnVision+ System (Agilent Technologies). All sections were counterstained with Hematoxylin and bright field images were captured at 20x or 40x magnification. Epithelial cells (100–200 cells) and CD3-immunoreactive T cells were also counted in both the apical portions and along the edges of the villi to assess intra-epithelial lymphocyte (IEL) infiltration of the small intestine and colon villi using methods adapted from previously reported studies [18, 19].

### Statistical analysis

All analysis was conducted using GraphPad Prism 7 (GraphPad Software, Inc, USA) and is presented as the mean ± standard error mean (SEM). Unpaired T-tests was performed to compare % weight changes, total body fat composition between Chow and HFD fed animals, CD3 immunoreactivity cells or eosinophils in representational sections of the small intestine or colon between Chow and HFD groups of macaque.

## Results

### HFD-induced changes in body composition

**Fig 1:**
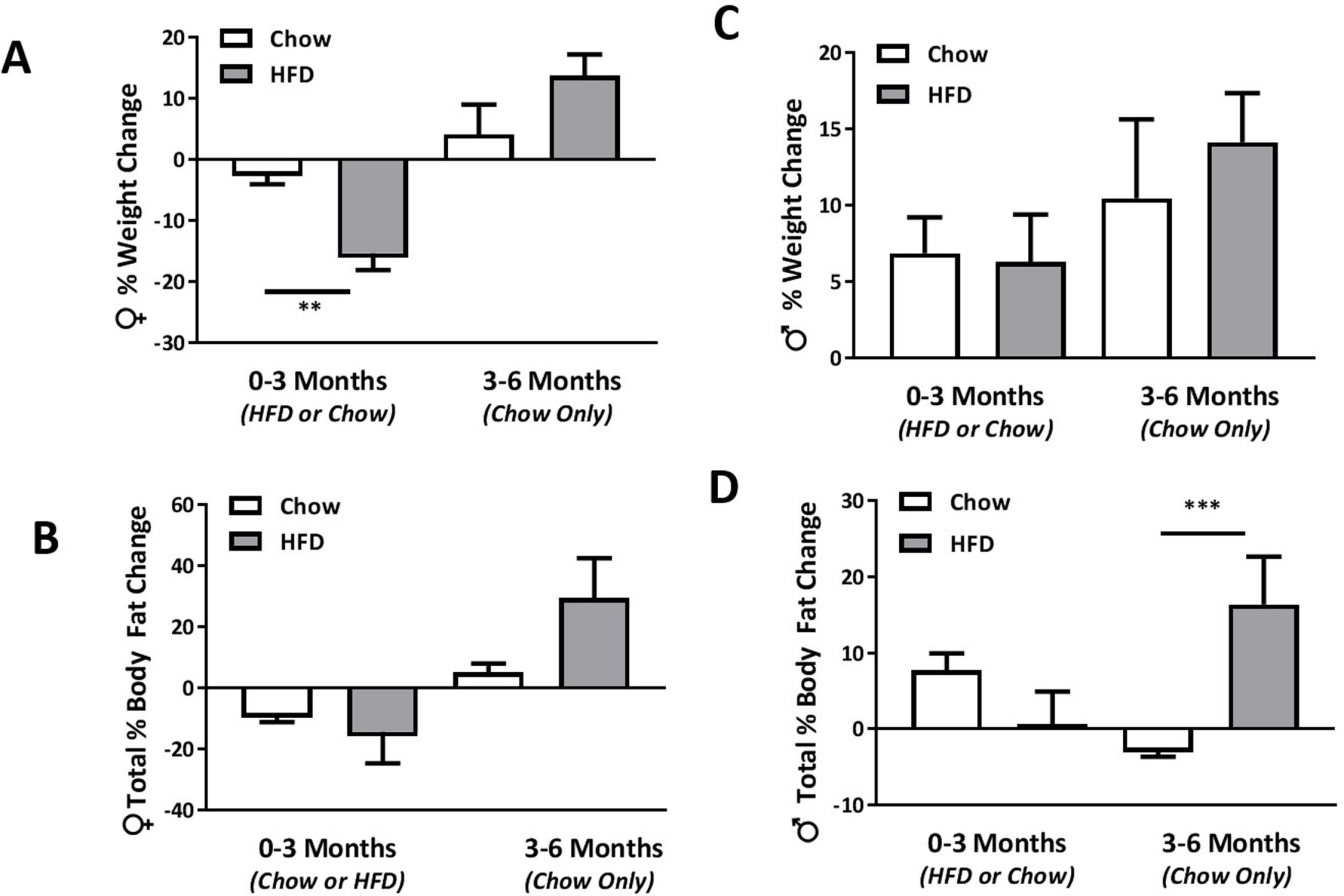
HFD consumption for 3 months resulted in significant weight loss in female macaques that was not evident in male macaques. Weights of female **(A)** and male **(C)** macaques was determined at baseline and prior to being placed on a custom HFD, while control counterparts remained on CHOW for 3 months. HFD animals were then reverted back to consuming the CHOW for three months (3-6months) and compared to CHOW-fed animals at the end of this remission period. Total % body fat composition of female **(B)** and male macaques **(D)** was examined using a DEXA at baseline, 3 months following consumption of HFD or CHOW (0–3 months) and at the end of the remission period (3–6 months). The difference in % change in total body weight and fat composition between HFD and CHOW at 0–3 and 3–6 was determined using unpaired student t-tests. **p<0.01, *p<0.05. Values are means ± SEM.

All females consuming the HFD (0–3 months) displayed 15% weight loss on average compared to the Chow-fed controls with three of the individual animals exhibiting > 20% weight loss as highlighted in Table 2 and Fig 1A. The changes in body weight correlated with reductions in total % body fat (Fig 1B), however these findings where limited due to the incomplete determination of body composition of the moribund females in the HFD group. In addition to body wasting, all HFD females examined at this time point demonstrated poor coat condition with marked whole body alopecia. Animals with > 20% chronic weight loss reached an experimental endpoint, and were removed from the protocol in accordance with AEC guidelines. The remaining female HFD animals were placed on the recovery diet (Chow-R) for a further 3 months. Assessment of body condition following this period, indicated that the HFD animals not only regained the weight that was lost during the previous 3 months, but gained significantly more weight than their control counterparts by the end of the study (Table 2 and Fig 1A). This change in total body weight correlated with a significantly higher total % body fat compared to control counterparts fed only Chow as highlighted in Fig 1B. All female animals also demonstrated full hair growth over this 3 month recovery period (results not shown).

**Table 2:** Summary of all female pigtail macaques used in the studies and diet-induced weight changes. Data presents the total % change in body weight of individuals from baseline following consumption of the HFD or Chow for 3 months (0–3 months). HFD animals were then switched back to consuming Chow for 3 months (3–6 months) while the control Chow animals continued to consume the Chow and the % change in body weight from 3 months was determined at the end of the remission period. † - Female animals that reached experimental endpoint during the studies and were removed from the study based on ethical guidelines.

Pigtail Macaques (*Macacca nemestrina*)	Age (Years)	Diet/ Group	% Weight Change 0–3 Months *(Chow or HFD)*	% Weight Change 3–6 Months *(Chow)*
**Females**	7.4	Chow-1	0.00	-10.81
	7.8	Chow -2	0.00	0.77
	6.7	Chow -3	0.00	2.24
	7.3	Chow -4	-6.85	39.12
	8.7	Chow -5	6.90	-2.26
	6.3	Chow-6	-4.76	*
	8.2	Chow -7	-6.10	3.25
	7.2	Chow -8	-6.94	6.57
	5.9	Chow -9	10.17	-8.92
	7.6	Chow -10	-11.39	7.43
**Mean ± SEM**			**-1.90 ± 2.11**	**4.15 ± 4.60**
**Females**	*12*.*3*	HFD -1*	*-31*.*82*	†
	13.7	HFD -2	-10.66	5.20
	*12*.*6*	HFD -3*	*-26*.*02*	†
	*13*.*6*	HFD -4*	*-20*.*00*	†
	14.2	HFD -5	-14.00	18.31
	7.7	HFD—6	-9.01	28.90
	8.5	HFD -7	-3.28	10.51
	8.4	HFD -8	-7.81	1.69
	8.2	HFD -9	-14.47	18.77
	12.1	HFD -10	-16.05	12.94
**Mean ± SEM**			**-15.31 ± 2.74**	**13.76 ± 2.90**

^†^ Female macaques that reached an experimental endpoint and were euthanised as part of the study.

^*^Female macaques removed from the study due to unrelated causes.

In contrast male macaques consuming the HFD for 3 months failed to show the expected increases in body weight compared to Chow fed animals (Table 3 and Fig 1C). Individual animals in this HFD group, either gained weight (1–21%) or displayed weight loss (4–11%) as shown in Table 3. These findings also correlated with lack of change in average total % body fat composition that was otherwise found to be significantly higher in control Chow-fed animals (Fig 1D). Close examination of HFD-males during this time (0–3 months) revealed that 3 out of the 10 animals displayed small focal areas of hair loss characteristic of excessive grooming. Switching the HFD male animals to standard chow for an addition 3 months however resulted in a significant weight gain (Table 3 and Fig 1C) and increases in total % body fat composition (Fig 1D) compared to control counterparts that remained on Chow during this period.

**Table 3:** Summary of all male pigtail macaques used in the studies and diet-induced weight changes. Data presents the total % change in body weight of individuals from baseline following consumption of the HFD or CHOW for 3 months (0–3 months). HFD animals were then switched back to consuming CHOW for 3 months (3–6 months) while the control CHOW animals continued to consume the CHOW and the % change in body weight from 3 months was determined at the end of the remission period.

Pigtail Macaques (*Macacca nemestrina*)	Age (Years)	Diet/ Group	% Weight Change 0–3 Months *(Chow or HFD)*	% Weight Change 3–6 Months *(Chow)*
**Males**	11	Chow-1	2.73	-2.09
	8.6	Chow -2	-1.30	-1.17
	6.5	Chow -3	3.33	8.89
	17	Chow -4	2.25	5.39
	10.2	Chow -5	1.54	-4.00
	4	Chow-6	6.25	44.17
	6.4	Chow -7	8.91	2.97
	4.5	Chow -8	5.36	16.79
	4.6	Chow -9	17.86	^*^
	4.5	Chow -10	21.82	23.27
**Mean ± SEM**			**-1.90 ± 2.11**	**4.15 ± 4.60**
**Males**	*7*	HFD -1	*7*.*19*	*2*.*01*
	6.3	HFD -2	1.11	17.25
	*7*.*3*	HFD -3	*9*.*38*	*4*.*95*
	*6*.*5*	HFD -4	*5*.*68*	*20*.*43*
	6.8	HFD -5	5.05	7.69
	7.4	HFD—6	20.69	^*^
	18.4	HFD -7	-4.12	15.81
	3.9	HFD -8	19.51	27.55
	7.7	HFD -9	-11.76	26.67
	8.5	HFD -10	-10.19	4.87
**Mean ± SEM**			**6.29 ± 3.11**	**14.14 ± 3.06**

^*^ Male macaques removed from the study due to unrelated causes.

### Post-mortem histopathological analysis

As previously mentioned, the three female animals removed from the protocol displayed >20% weight loss, severe emaciation and poor body condition marked with diffuse hair loss. Histopathological examination of skin necropsy samples from these animals revealed mild follicular atrophy and telogen effluvium (resting hair follicle) with no obvious inflammation of the skin or presence of infectious organisms. Macroscopic examination of the intra-abdominal cavity revealed severely distended bowels wrapped in fat in all animals examined, otherwise not evident in control animals taken from unrelated studies and used as Chow controls in the present studies (results not shown).

**Fig 2:**
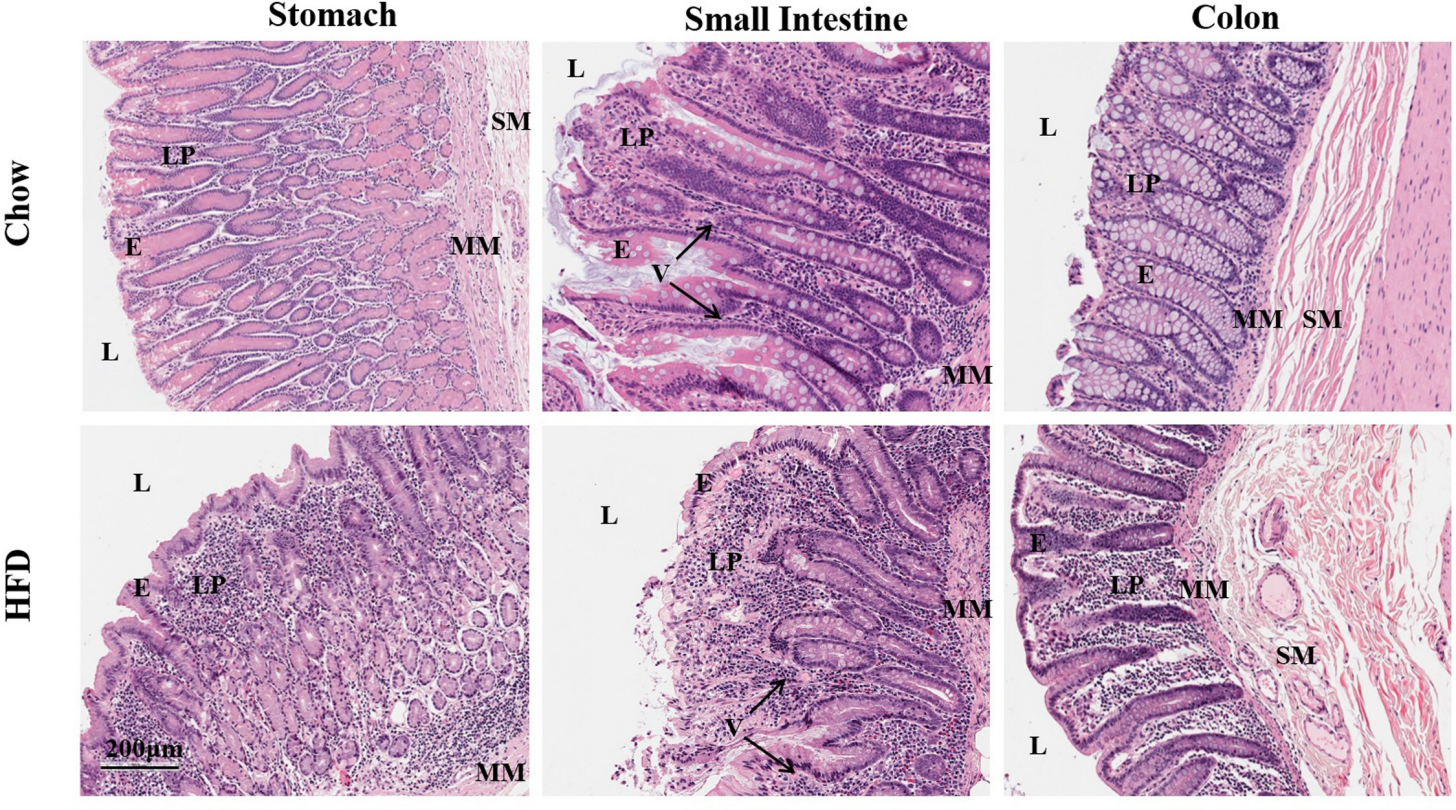
HFD-induced histopathological changes in female macaques indicating severe inflammation throughout the GIT. Representative sections of inflamed mucosa in the stomach, small intestine and colon of HFD-fed female macaques (lower panel) compared to control Chow counterparts. Consumption of the HFD resulted in thickening and immune cell, mainly lymphocyte, infiltration of the laminia propria while the muscularis mucosae and submucosa remained largely intact. The intestinal villi were flat as a result of immune cell infiltration. L: Lumen; E: epithelium; LP: Lamina propria; MM: muscularis mucosae; SM: submucosa; V: villi.

Histopathological examination of stomach segments from these animals revealed marked mucosal and laminia propria (LP) hypertrophy that was due to inflammatory cell infiltrates most likely of lymphocytic origin (Fig 2). Similarly microscopic examination of the small intestine, revealed severe villi blunting and atrophy with varying crypt depth. The villi and the laminia propria were thickened by inflammatory cells, what appeared to be of largely lymphocytic and granulocytic origin that did not extend into the submucosa (Fig 2). Unlike the small intestine, pathological changes in the large intestine of these HFD animals was discontinuous with several segments appearing normal and comparable to Chow-fed control tissue. In affected regions, there was gross changes to the mucosa marked by dense immune cell infiltrates, sever loss of goblet cells, crypt distortion and luminal surface changes as shown in Fig 2. No evidence of bacterial and other infection was found in the stomach, small intestine and colon of these HFD-fed female subjects.

### Characterisation of immune cell infiltrates in the gastrointestinal tract

**Fig 3:**
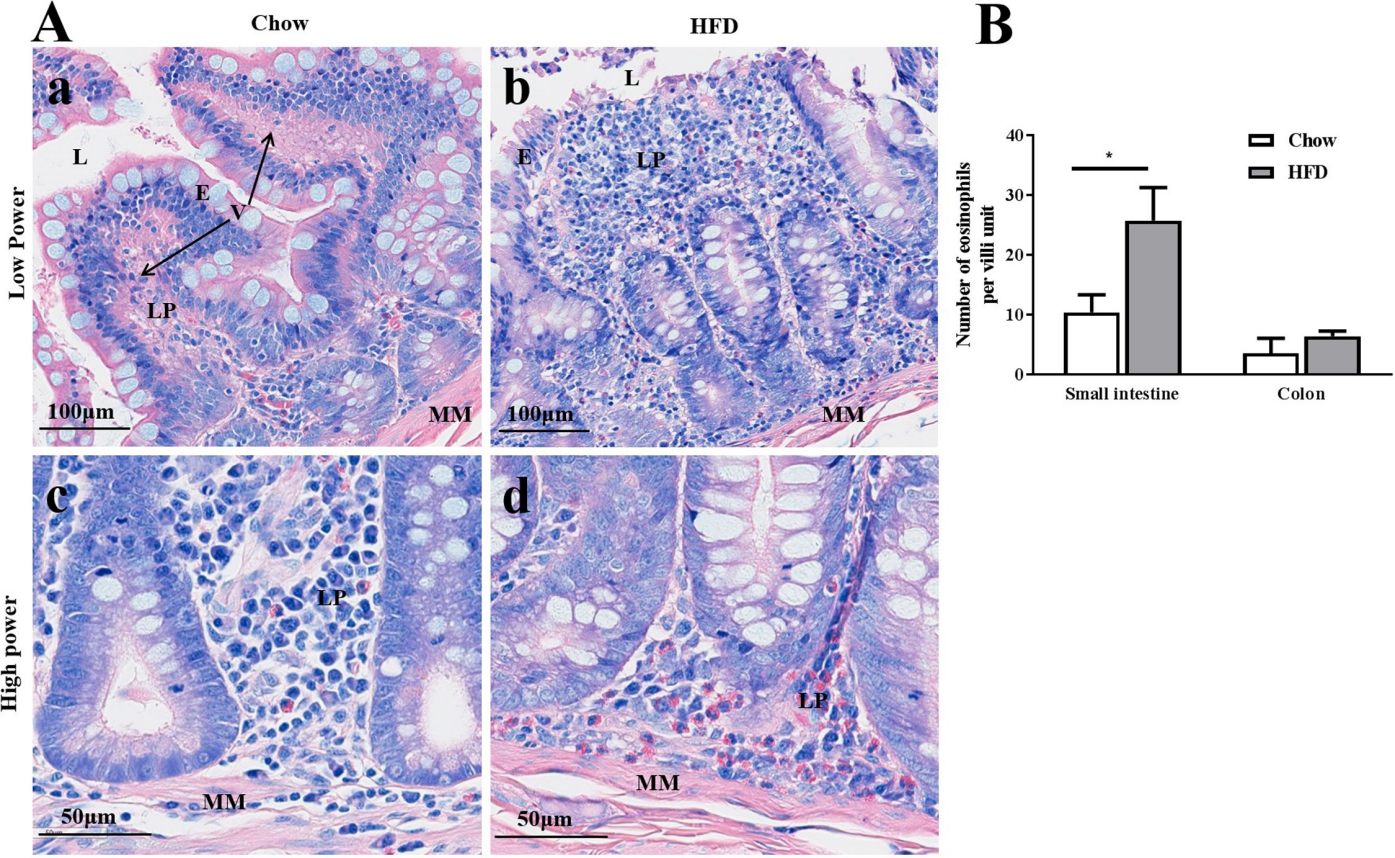
HFD-induced eosinophils infiltration of the small intestine mucosa of female animals. **(A).** Representative sections of both normal chow-fed **(a, c)** and inflamed HFD-fed **(b, d)** small intestine mucosae respectively showing the infiltration of eosinophils. High power magnification showed clustered eosinophils at the basal zone of mucosae in inflamed tissue **(d)**, compared with the scattered distribution of eosinophils in Chow-fed animals **(c)**. **(B)**. Quantification and comparison of eosinophil numbers between Chow and HFD small intestine and colon. Values are means ± SEM. *P<0.05. L: Lumen; E: epithelium; LP: Lamina propria; MM: muscularis mucosae; V: villi.

Additional histological and immunological staining was conducted in the stomach, small intestine and colon of HFD to better characterise the origin of the immune cell infiltrates. Sections of the small intestine stained with Giemsa May-Grünwald (MG) demonstrated significant infiltration of the lamina propria region with clusters of eosinophils at the basal mucosal zone of HFD animals (Fig 3A(b)) that was otherwise sparsely scattered in Chow-fed control animal tissue (Fig 3A(a)). Quantitation of eosinophilic infiltration of the gastric wall demonstrated that infiltration was specific to the small intestines, and remained unchanged in the colon of HFD animals compared to control animals (Fig 3B).

**Fig 4:**
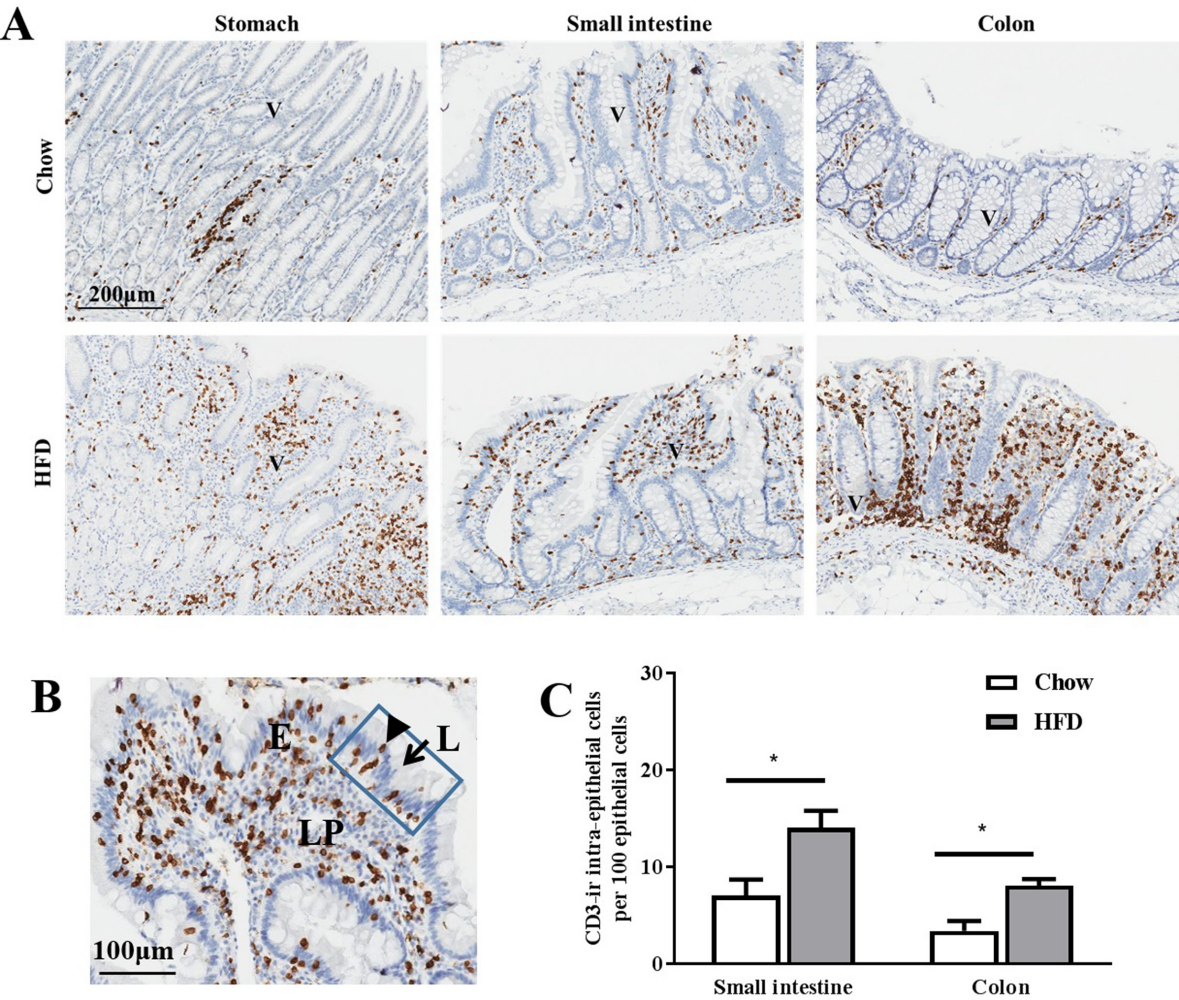
Diet-induced intra- and extra- epithelial CD3+ immunoreactivity in the gastrointestinal tract of female animals. **(A).** Immunohistochemical analysis of CD3-ir (immunoreactivity) in representational sections of stomach, small intestine and colon obtained from Chow and HFD-fed animals. **(B)**. Magnification of villi of inflamed small intestine obtained from HFD animals showed increases in CD3 immunoreactive T cells (CD3-ir) that was not restricted to only epithelial cells of small intestine and was also found in lamina propria. **(C).** Comparison of the amount of CD3-ir intra-epithelial T cells in the small intestine and colon of Chow vs HFD groups. Values are means ± SEM. *P<0.05. L: Lumen; E: epithelium; LP: Lamina propria. The rectangle box indicated the part of epithelium where epithelial cells (arrow) and CD3-ir T cells (arrowhead) was counted.

Immunohistological staining for CD3, a marker of T-cells, revealed significantly higher numbers and severe accumulation of CD3 positive cells (CD3+) in the lamina propria of HFD-stomach, small intestine and colon compared to Chow-fed control animals (Fig 4A). Closer examination and quantitation of the distribution of CD3+ cells in the mucosal villi suggested even though there was increased lymphocyte infiltration of the intraepithelial layers of the small intestine and colon following consumption of the HFD, this was not restricted to epithelial cells and was seen disbursed throughout the entire lamina propria (Fig 4B and 4C).

**Fig 5:**
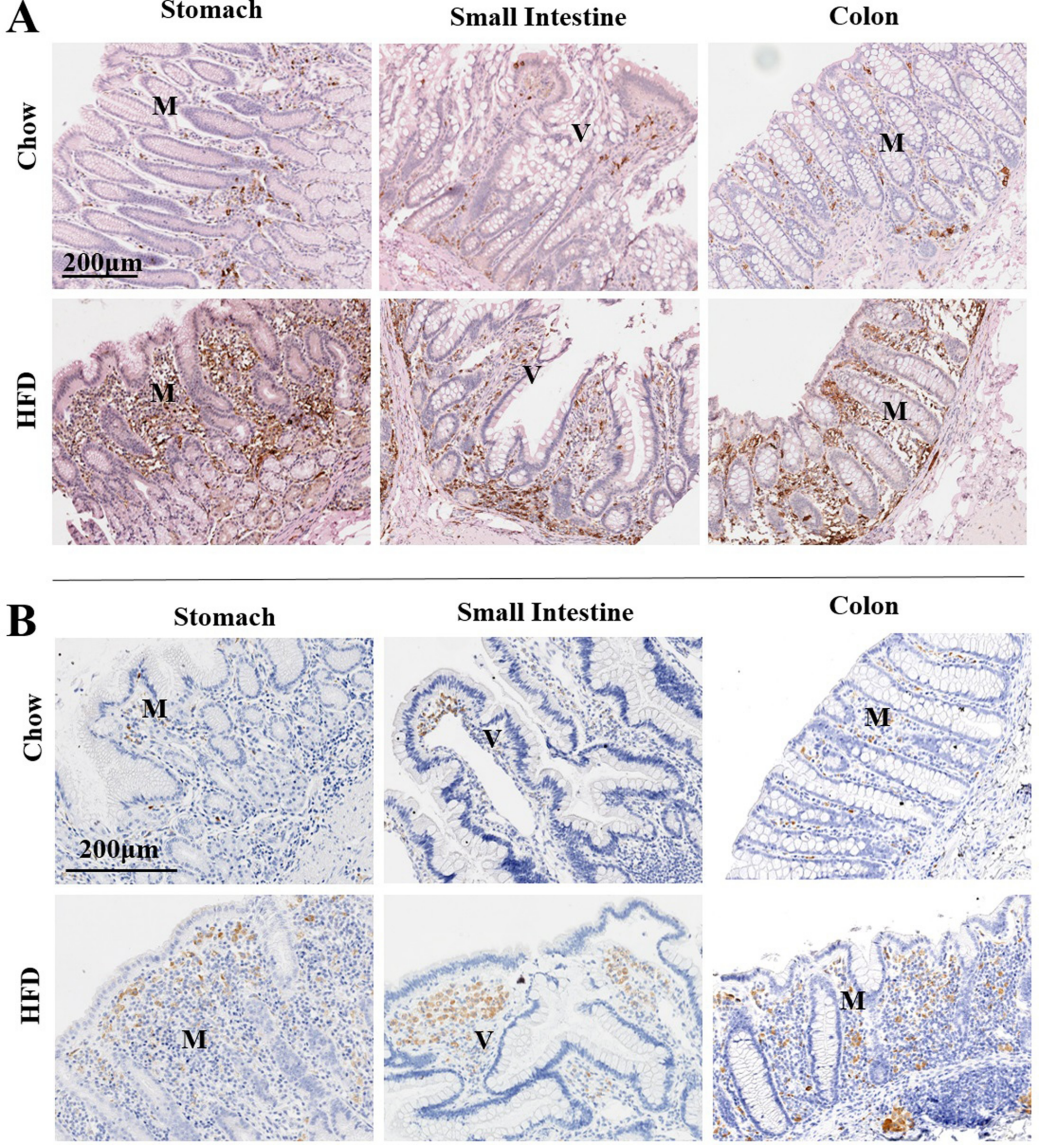
**(A) Changes in gastrointestinal CD11C-immunoreactivity in response to diet-induced inflammation.** Immunohistochemical analysis of CD11C, a marker of dendritic cells in addition to monocytes/macrophages, neutrophils and nature killer cells in representational sections of stomach, small intestine and colon of CHOW (top panel) or HFD (lower panel) counterstained with Hematoxylin from female macaques. **B. Increased CD68 immunoreactivity throughout the gastrointestinal tract of HFD-fed animals.** Immunohistochemical analysis of CD68, a marker of macrophages in representational sections of stomach, small intestine and colon of Chow (top panel) or HFD (lower panel) fed female animals counterstained with Hematoxylin from female macaques.

**Fig 6:**
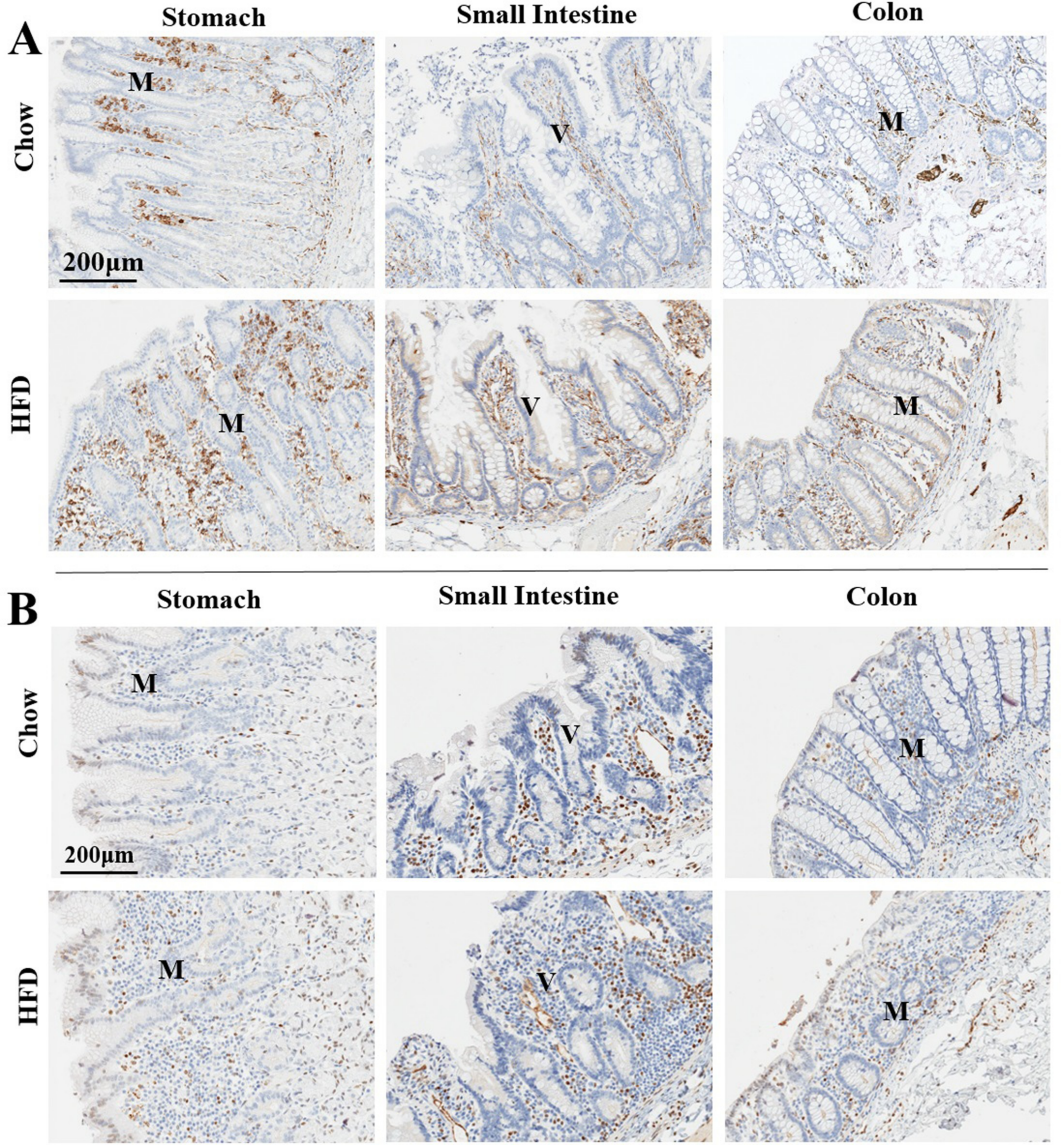
**A. Recruitment of CD56 immunoreactive cells to the gastrointestinal tract in response to HFD in female animals.** Immunohistochemical analysis of CD56, a marker for natural killer cells, T cell subtypes and monocytes in representational sections of stomach, small intestine and colon of Chow- (top panel) or HFD- (lower panel) fed female animals counterstained with Hematoxylin. **B. Dietary manipulations did not alter recruitment of CD8 immunoreactive cells to the gastrointestinal tract of female macaques.** Immunohistochemical analysis of CD8, a marker of cytotoxic T cells in representational sections of stomach, small intestine and colon obtained from Chow-(top panel) or HFD- (lower panel) fed animals counterstained with Hematoxylin from female macaques.

The intensity of staining for CD11C (Fig 5A) and CD68- immunoreactive cells (Fig 5B), were also increased in the lamina propria and basal areas of the mucosae in all HFD tissues evaluated compared to Chow-fed control counterparts suggesting recruitment of dendritic cells, monocytes/macrophages, neutrophils and natural killer cells to stomach, small intestine and colon of HFD females. In contrast, the density of immunoreactivity for CD56, a more selective marker for nature killer cells (Fig 6A) and CD8, a marker for cytotoxic T cells (Fig 6B) were not altered between HFD and Chow fed groups.

## Discussion

To investigate a NHP model of diet induced-obesity, Southern pigtail macaques were placed on a HFD formulated to the standard TAD primate diet [3, 4]. Consumption of this diet for 3 months did not result in the expected increase in body weight but instead induced chronic weight loss, significant body wasting, alopecia, malabsorption, protein-losing enteropathy and chronic inflammation throughout the gastrointestinal tract in a sex-specific manner. All HFD-induced clinical features were fully resolved within 3 months of discontinuing this diet and suggests the adverse effects observed in our studies, were a direct consequence of the diet change.

Disturbances in gastrointestinal health have been reported to be the main cause of illness and mortality in captive-bred macaque colonies [10–13]. Close examination of the medical history of the colony used in our studies also indicated the most common health issues diagnosed was idiopathic gastrointestinal disturbances defined to be a form of irritable bowel disease (IBD). The strategy employed to reduce the occurrence of IBD in the colony was to employ a low FODMAP (fermentable oligosaccharides, disaccharides, monosaccharides and polyols) diet to reduce and control the occurrence of irritable bowel syndrome (IBS) in humans [20]. The standard primate diet (Chow) used to maintain the entire colony, and used as the control diet in our studies, was based on a low FODMAP diet. This strategy was found to greatly reduce, but not completely eliminate, the spontaneous occurrence of gastrointestinal disturbances in the colony. A comparison of the ingredient constituents between the HFD and Chow revealed addition of macronutrients such as grains (wheat, bran and pollard), dairy (skim milk and casein) in addition to fat (beef tallow) into the HFD that was originally omitted from the regular Chow. The HFD was also manufactured with a considerably lower protein content compared to Chow or TAD. As such, the food-driven inflammation and symptomologies observed in our studies, were most likely caused by the introduction of the HFD to the colony with an existing history of gastrointestinal disturbances and sensitivities. The protein deficiency of the diet may have further exacerbated weight loss / wasting.

This study has important implications for the husbandry of pigtail macaque colonies. Up to the age of 4 years old both male and female pigtail macaques typically gain on average 1kg/year on standard primate diet [21]. After this age, females continue to gain 1kg/year (0.25kg/3months) while males gain up to 2kg/year (0.5kg/3months) [21]. Despite husbandry conditions influencing these findings, the consumption of our HFD should have resulted in weight gain in excess of these regular growth-related changes. Even though Southern pigtail macaques have not been previously used to establish experimental models of diet-induced obesity, studies using male rhesus macaques fed the TAD primate diet demonstrated an average 20% weight gain over a 4-month period [3]. Different from these published studies was the fact we did not supplement the diet with the use of fructose drinks and the animals were not restricted with physical activity, however we do not feel this was the basis for the lack of weight gain in males or the significant body wasting observed in female macaques. All female macaques that consumed the HFD for three months displayed 15% weight loss on average, with three exhibiting 20–30% total weight loss.

A sexual dymorphic allergic response has been known for quite some time, which was reinforced by a recent meta-analysis study that highlights woman are typically prone to developing food allergies and intolerances compared to males [22]. Females challenged with specific allergens, pathogens and vaccines also typically mount a higher immune response compared to males [23]. Sex hormones such as estrogen have been proposed to directly bind and regulate the activity of immune cells and likely to be involved in the development of IBS or inflammation of the gut in females compared to men [24]. Sex-specific differences in the gut microbiome has also been documented [25] and gut dysbiosis induced through dietary changes could intern impact inflammatory processes in the gut [26]. These factors could partly explain the predisposition and hyper-immune response of females to the HFD, as a healthy state could be fully restored within 3 months following removal of the HFD, to levels higher than controls, confirms a direct effect of diet.

Importantly the clinical features observed in our studies were of delayed onset. The earliest detection of any noticeable changes in body condition, including hair loss, only became evident following chronic exposure of the animals to the HFD for at least 2 months prior the earliest indicator being hair loss. This hair loss was not due to excessive grooming or plucking which is usually focally restricted to specific areas. Microscopic histopathological analysis of skin and hair revealed mild follicular atrophy and telogen effluvium (resting hair follicle) that was not associated with local inflammation or a pathogenic cause. Under conditions of extreme metabolic stress, malnutrition and low protein, hair is known to prematurely enter the telogen resting phase and result in notable hair loss. As previously mentioned the HFD was also formulated with lower protein content that may have also contributed to hair loss and the diagnosis of protein-losing enteropathy and malabsorption in the animals. Elimination of the HFD and placing the animals on regular Chow supplemented with extra protein fully restored body condition and enabled full hair regrowth within 3 months.

There are some similarities of our results with another report of captive rhesus macaques [15]. In that case the colony was reported to be exhibiting chronic diarrhoea of non-infectious origin diagnosed primarily as celiac disease that could be fully reversed by eliminating gluten from the diet. As mentioned previously our HFD also introduced animals to gluten in the form of wheat, bran and pollard, and like these studies, we also found an increase in the numbers of intraepithelial lymphocytes in the small intestine and colon villi that is typically used for diagnosis of celiac disease [27]. However, the animals in this study did not present with the full spectrum of clinical features associated with celiac disease including chronic diarrhoea, rashes and distension of the stomach. Our analysis of serum from these animals also failed to reveal elevated levels of anti-glandin and transglutaminase antibodies (results not shown), that was also correlated with the clinical symptoms reported in the NHP model of gluten sensitivity [15].

## Conclusion

In establishing a model of HFD-induced obesity, we have uncovered a delayed food hypersensitivity-mediated inflammatory response that caused life-threatening complications in female captive Southern pigtail macaques that could be fully reversed by eliminating the offending food sources. This diet–induced gastroenteropathy could not be classified under any particular cluster of gastrointestinal inflammatory diseases (i.e. inflammatory bowel-, celiac- disease or idiopathic colitis), but it shared many overlapping clinical features. Dietary interventions are commonly used to create models of diet-induced obesity and this usually involves the introduction of food sources that are atypical of the standard primate diet. We have shown that the ingredients used to create these diets must be carefully reviewed and cross-referenced with not only the standard primate diet, but also the overall dietary and clinical history of the captive colony. While other forms of food sensitivities and intolerances can result in immediate noticeable symptoms, this was not the case in our studies and resulted in late diagnosis. Diet-related changes should be carefully reviewed and taken into consideration when diagnosing any gastrointestinal disorders in captive macaques.
